# Do overweight and obese individuals demonstrate impaired thermoregulatory adaptation to six weeks of studio cycling training: A pilot study

**DOI:** 10.1186/2046-7648-4-S1-A115

**Published:** 2015-09-14

**Authors:** Steve H Faulkner, Kieran Menon, Thomas M Hood, Jamie K Pugh, Myra A Nimmo

**Affiliations:** 1 School of Sport, Exercise and Health Sciences, Loughborough University, Loughborough, UK

## Introduction

High intensity interval training (HIIT) is effective at improving health markers in groups at risk of cardiovascular and metabolic disease. Studio cycling may offer a platform for HIIT in the community, however little is know about how increased adiposity influences thermal control during HIIT and the effect this may have on overweight patients who are new to exercise. The purpose of this pilot study was to investigate the thermal adaptation in response to studio based group HIIT in sedentary overweight adults.

## Methods

Eight, overweight, physically inactive (86.1(10.6) kg; $\overset{\cdot }{\text{V}}{\text{O}}_{2\text{max}}$ 27.1(4.7) mL.kg.min^-1^; BSA 1.97(0.16) m^2^; 31.8(2.4) %body fat; <1.5 hr.wk^-1 ^of physical activity) otherwise healthy volunteers completed six 30 minute familiarisation sessions followed by 6 weeks of supervised studio cycling 3 times per week. All sessions were completed in a ventilated but non-temperature controlled group fitness studio. Thermal measures were completed in weeks 1, 3 and 6 for all participants. Participants were weighed in minimal clothing before and after exercise, which was corrected for fluid intake to determine sweat loss. During the session, wireless thermistors were attached to the skin for the calculation of mean skin temperature (${\bar{\text{T}}}_{\text{sk}}$) and heart rate was recorded for intensity assessment. All data presented as mean (SD).

## Results

Mean ambient temperature across all weeks was 19.6 ± 1.7 °C. The mean and peak exercise intensity equated to 83(4)% and 9(4) % of HR_max _respectively with no differences between weeks. Body fat was reduced by 13% following training (pre: 31.8(2.4) %; post 27.5(4.5) % (p < 0.05), but no change for BMI, body surface area or body surface area:mass. There was a significant effect of time (p < 0.0001) and an interaction (time × week, p < 0.0001) on ${\bar{\text{T}}}_{\text{sk}}$. ${\bar{\text{T}}}_{\text{sk}}$ was lower for week 3 compared to week 1 from 25 minutes onwards (p < 0.05) and compared to week 6 from 15 minutes onwards. ${\bar{\text{T}}}_{\text{sk}}$ was higher in week 6 compared to week 1 from 30 minutes (p < 0.05). There was a significant effect of time (p < 0.0001) and an interaction (time × week, p < 0.0001) on $\text{Δ}{\bar{\text{T}}}_{\text{sk}}$. $\text{Δ}{\bar{\text{T}}}_{\text{sk}}$ was lower for week 3 compared to both week 1 and week 6 from 15 minutes onwards (p < 0.05). $\text{Δ}{\bar{\text{T}}}_{\text{sk}}$ remained higher in week 6 compared to week 1 from 35 minutes (p < 0.05). ${\bar{\text{T}}}_{\text{sk}}$ was moderately correlated to sweat loss corrected for body surface area (r=.51, p < 0.05). There was a trend towards a correlation between ${\bar{\text{T}}}_{\text{sk}}$ and body fat percentage (r=-.40, p = 0.120). Sweat loss corrected for BSA tended to increase from week 1 to 6 (260.9(0.15) mL.m^2 ^vs 321.1(93.5) mL.m^2^; p = 0.183) and was correlated to mean Tsk (r=.36, p < 0.01) and body composition (r=-.55, p < 0.05).

## Discussion

The present data suggest that there may be some impairment of thermoregulatory adaptation in response to regular indoor cycle training in overweight and obese individuals. The decline in ${\bar{\text{T}}}_{\text{sk}}$ in week 3 compared to week 1 prior to an elevation in ${\bar{\text{T}}}_{\text{sk}}$ in week 6 suggests that the thermal adaptation response evident in healthy lean individuals may be hindered in overweight. It is likely that this response is in someway due to elevated adipose tissue in these individuals. Increased adiposity will impair conductive heat exchange from core to skin during exercise and is further compounded by the low specific heat of fat compared to skeletal muscle. This will therefore limit changes in ${\bar{\text{T}}}_{\text{sk}}$ and heat loss during initial training bouts until body fat is reduced, exposing these individuals to a potentially higher risk of heat illness.

## Conclusion

Care should be taken when overweight or obese individuals begin exercise training, as it is possible that they may experience greater thermoregulatory strain in the initial phases of training before heat acclimation begins to occur. Further work should consider the effect that increasing exercise prescription to overweight and obese populations has on the risk of heat related complications.

